# The handbook of physics in diagnostic imaging

**Published:** 2008

**Authors:** K. N. Govinda Rajan

**Affiliations:** Professor of Medical Physics, Department of Physics, PSG College of Technology, Coimbatore - 643 004, India. E-mail: kngrajan@gmail.com

‘The Handbook of Physics in Diagnostic Imaging’, authored by Dr. Roshan Livingstone and published by B. I. Publications Pvt. Ltd., Chennai, covers the important diagnostic modalities in a summary fashion. The modalities covered under x-ray diagnosis are radiography, fluoroscopy, tomography, digital radiography, and special radiography. This is followed by MRI and ultrasound and two chapters devoted to radiation protection and quality assurance.

The first chapter on basic physics defines radiation, atom, radioactivity and radiation protection-quantities-and units. The topic of basic dose quantities relevant to radiology is briefly discussed, and patient dose monitoring (entrance skin dose, dose area product, etc.) is mentioned in the chapter on radiation protection. A section on patient dosimetry would have made the book more useful for practicing physicists or technologists.

The second chapter deals with x-ray tubes, x-ray generators, x-ray production and properties, and few of the safety interlocks.

The third chapter on interaction of radiation with matter discusses the basic x-ray interactions and how the transmitted fluence profile influences the image contrast at high and low kV.

The fourth chapter on radiation scatter deals with the effect of scatter radiation on patient dose and the image quality and the function of grid in radiography.

The fifth chapter on film is a little elaborate compared to other chapters. It discusses the film structure, its properties, its development procedure, definition of film speed, and the use of screen-film for patient dose reduction. Chapter 6 on radiographic image quality is very short, and it briefly describes subject/image contrast, resolution, and unsharpness.

The next three chapters deal with radiographic image quality, image intensifier (II) fluoroscopy, and Computed Tomography (CT). The chapter on image quality describes the factors that influence contrast, resolution, and geometric unsharpness. The chapter on fluoroscopy is a summary of various fluoroscopic procedures in practice, namely, direct fluoroscopy (now almost in disuse), image-intensified fluoroscopy, cine and serial radiography. The chapter on CT discusses various generations of CT, their differences, the principles of image formation, and the artifacts arising in CT imaging. A very brief introduction on CT dose is also given at the end of the chapter.

The chapter on digital radiography deals with the principles of CR (computed radiography) systems and the advantages of these systems over analog radiography or screen-film radiography. The technology involved in the design of storage phosphor and the readout systems is also explained in a simple way. The last section discuses the concept of Picture Archiving and Communication System (PACS).

The chapter dealing with special radiography only lists the various types of equipment, like the dental radiography unit, portable radiography unit, and the mammography unit. Considering the importance of mammography, a separate chapter could have been devoted to mammography.

The chapter on radiation protection describes radiation risks in terms of deterministic and stochastic effects and the method of determining effective dose received by a radiation worker from the doses to the individual organs and their relative sensitivities. The dose limits for the radiation worker and the public, as recommended by the regulatory bodies (e.g., ICRP), are also mentioned in this chapter. The chapter also mentions about personal monitoring, and there is a discussion of free air ionization chamber. A brief discussion of the regulatory agency in the country (the Atomic Energy Regulatory Board) and regulations on safe use of radiation is also done in this chapter.

The next two chapters deal with ultrasound and magnetic resonance imaging that do not involve the use of ionizing radiation and hence the safety concerns are much less compared to x-ray imaging. The principles of image formation using sound waves in ultrasound and radio frequency (RF) waves and nuclear resonance in magnetic resonance imaging are dealt with in this section. The last chapter on quality assurance is too brief and is confined to a handful of QA tests.

Since the book deals with all the topics in a summary fashion, it can serve as a desktop reference for x-ray technologists. Hence the book is a useful addition to the cause of teaching or practicing diagnostic imaging physics at the technologists′ level.

